# Supplementary material for: Reconciling Mining with the Conservation of Cave Biodiversity: A Quantitative Baseline to Help Establish Conservation Priorities
Source: PLoS One. 2016 Dec 20;11(12):e0168348. doi: 10.1371/journal.pone.0168348 (PMC5173368; doi:10.1371/journal.pone.0168348)
Supplement: S1 Dataset — (ZIP) [file pone.0168348.s002.zip › Taxa/Serra Norte/SN_2013/N4WS/Lista N4WS-54.pdf]

CAVIDADE N4WS-0054

Classe	Ordem	Fam/Outros	Gên/Outros	Espécie	S-U	U-S	Zon.
Arachnida	Amblypygi	Phrynidae	*Heterophrynus*	*longicornis*	1	1	E
Arachnida	Araneae	Drymusidae		jovem	X		E
Arachnida	Araneae	Filistatidae		sp.1		X	E
Arachnida	Araneae	Pholcidae	*Mesabolivar*	*eberhard*	X	X	E
Arachnida	Araneae	Scytodidae	*Scytodes*	*eleonorae*		1	P
Arachnida	Araneae	Theridiosomatidae	*Plato*	sp.1	X	X	P
Entognatha	Collembola	Paronellidae		sp.4	X		E
Insecta	Blattodea			jovem	1		E
Insecta	Diptera	Cecidomyiidae	Cecidomyiinae	sp.	X		E
Insecta	Diptera	Ceratopogonidae		jovem	X		E
Insecta	Diptera	Psychodidae	*Evandromyia*	*saulensis*		X	P
Insecta	Diptera	Psychodidae	*Micropygomyia*	*pilosa*		X	P
Insecta	Diptera	Tipulidae	Tipulinae	sp.	X	X	E/P
Insecta	Heteroptera	Reduviidae	Emesinae	jovem		1	E
Insecta	Hymenoptera	Formicidae	*Camponotus*	sp.1	X	X	E/P
Insecta	Hymenoptera	Formicidae	*Gnamptogenys*	*striatula*		X	E/P
Insecta	Hymenoptera	Formicidae	*Nylanderia*	sp.3		X	E
Insecta	Hymenoptera	Formicidae	*Solenopsis*	sp.2		X	E
Insecta	Orthoptera	Phalangopsidae	*Paraclodes*	sp.	8	3	E
Insecta	Orthoptera	Phalangopsidae	*Phalangopsis*	sp.		3	E
Insecta	Psocoptera	Psyllipsocidae		jovem		X	E
Amphibia	Anura	Strabomantidae	*Pristimantis*	cf. *fenestratus*	1		E
Mammalia	Rodentia	Cricetidae	*Rhipidomys*	sp.		1	P
